# Editorial: Healthy ageing, social psychiatry of older adults and family caregivers

**DOI:** 10.3389/fpsyt.2026.1791727

**Published:** 2026-02-05

**Authors:** Ivy Yan Zhao, Yuka Kotozaki, Nan Jiang

**Affiliations:** 1 School of Nursing, The Hong Kong Polytechnic University, Hong Kong, Hong Kong SAR, China; 2 School of Medicine, Iwate Medical University, Morioka, Japan; 3 Division of Orthopaedics and Traumatology, Department of Orthopaedics, Nanfang Hospital, Southern Medical University, Guangzhou, China

**Keywords:** chronic disease, family caregiver, healthy ageing, household composition, meaning in life, social integration, social psychiatry

## Introduction

Population ageing is reshaping our societies worldwide. Social psychiatry provides a critical lens for understanding how family relationships, community participation, cultural beliefs, and care arrangements shape mental health in later life. The articles in this Research Topic illuminate these dynamics across diverse contexts, from nursing homes and “empty nest” households to spousal caregiving and chronic disease management, and highlight the parallel vulnerabilities of family caregivers.

## Dignity, attitudes toward death, and meaning in late life

The qualitative study by Sunzi et al. on older adults’ perceptions of dignity in nursing homes foregrounds an issue that is central to healthy ageing yet frequently neglected in practice. Residents’ sense of dignity is profoundly shaped by their physical dependence, the quality of staff–resident interactions, and opportunities for meaningful social engagement. Where autonomy, respect, and personhood are upheld, residents exhibit better psychosocial adjustment; when dignity is compromised through neglect, infantilisation, or lack of privacy, emotional distress and withdrawal are common. This work highlights that mental health promotion in nursing homes should be embedded in everyday relational and organisational practices, not merely in the availability of clinical services.

A complementary cultural perspective emerges from the study of positive death attitudes and psychological well-being among Buddhist Thai older adults by Glushich et al. Drawing on the concept of the four immeasurable: loving-kindness, compassion, empathetic joy, and equanimity, the authors show that positive beliefs about death are associated with enhanced well-being, mediated through prosocial values. Rather than viewing death solely as a source of fear, the study suggests that accepting mortality within a spiritual framework can foster psychological resilience and a sense of meaning in late life. This suggests that culturally adapted, mindfulness- and compassion-based approaches may be particularly valuable in promoting healthy ageing in religious and spiritual communities.

## How physical health and social integration shape later life mental health

A nationwide cohort study on cardiovascular disease (CVD) and depressive symptoms by He et al. demonstrates a longitudinal, bidirectional association in mid-to-late life, with CVD exerting a stronger influence on subsequent depression than vice versa. These findings strongly support integrated care models in which cardiovascular and mental health are addressed together. Moreover, social integration is now recognised as a major determinant of late-life mental health. A study by Deng et al. on social integration and depression risk among middle-aged and older Chinese adults with multiple chronic conditions introduces important nuance to discussions of “social connectedness.” Economic integration and community integration were linked to lower depression risk, whereas relational integration was associated with higher depression risk. This counterintuitive finding suggests that some relationships, which perhaps marked by conflict, obligation, or stigma may be emotionally burdensome rather than protective. The heterogeneity across demographic groups, supported by sensitivity and subgroup analyses, underscores the need for more granular approaches that distinguish between supportive and stressful forms of social connection.

## Family support, household composition and ageing-in-place

Family emotional support and household remain central to the experience of ageing-in-place. A study by Wu et al. proposing an emotional support model in ageing families shows that being emotionally supported by relatives and engaging in social activities bolster older adults’ self-perceptions and positive mental health. Ageing attitudes and interaction patterns emerge as critical pathways through which family dynamics influence psychological well-being. These findings argue for integrating family-centred interventions into gerontological and psychiatric practice, for example, by promoting constructive communication, shared activities, and positive narratives of ageing within families.

Household composition is also a key social determinant. Guo et al.‘s cross-sectional study from Guangdong on disability and cognitive impairment among empty nesters versus non-empty nesters reveals higher prevalence of both outcomes among empty nest older adults. Risk factors differ by household type: while low/middle income, polypharmacy, family support, and depression are common contributors to disability, anxiety is uniquely important among empty nesters. Similarly, literacy, residence, and depression are shared risk factors for cognitive impairment, whereas polypharmacy is specific to empty nesters and coronary heart disease to non-empty nesters. These findings underscore that interventions must be tailored to living arrangements; policies and services designed around an assumed co-resident family caregiver may leave empty nesters particularly vulnerable.

## Attention to family caregivers’ mental health is urgently needed

Family caregiving has become a cornerstone of long-term support. However, a CHARLS-based study by He et al. on spousal care shows that caregiving has a pronounced negative impact on mental health, especially for female and rural caregivers. Reduced exercise and increased non-food expenditure are identified as mechanisms linking caregiving to psychological strain. This challenges the romanticised notion of family care as purely beneficial and underscores the need for policies that provide respite, financial support, and accessible mental health care to spousal caregivers.

A cross-sectional study on social alienation among caregivers of stroke patients by Xu et al. further highlights the relational and societal dimensions of caregiver distress. Caregivers, particularly those living with the patient, divorced or widowed, bearing heavy care burdens, with low social support and high loneliness experience high levels of alienation. Social psychiatry’s emphasis on social inclusion and community support is particularly relevant here: targeted, individualised interventions, caregiver education, peer support networks, and integration of caregiver needs into stroke care pathways are essential.

## Conclusion

Taken together, this Research Topic reaffirm the central premise of social psychiatry that mental health in older age cannot be understood in isolation from social structures, cultural meanings, and interpersonal relationships. The included studies illustrate how experiences of dignity, attitudes toward death, social integration, family support, caregiving burden, and household composition intersect with physical illness and functional limitations to influence psychological outcomes in later life.

By foregrounding both older adults and family caregivers, this Research Topic moves beyond deficit-oriented narratives of ageing and caregiving, and instead highlights the dynamic, relational, and context-dependent nature of mental health in ageing societies. These insights call for policies and interventions that extend beyond clinical treatment to encompass social inclusion, family dynamics, and community-based support systems. In doing so, this Research Topic contributes to a more holistic and socially grounded understanding of healthy ageing.

